# NMR-based metabolomic profiling of *Peganum harmala* L. reveals dynamic variations between different growth stages

**DOI:** 10.1098/rsos.171722

**Published:** 2018-07-18

**Authors:** Yinping Li, Qing He, Zhufeng Geng, Shushan Du, Zhiwei Deng, Eerdun Hasi

**Affiliations:** 1 College of Chemistry, Beijing Normal University, Beijing 100875, China; 2 Analytic and Testing Center, Beijing Normal University, Beijing 100875, China; 3 State Key Laboratory of Earth Surface Processes and Resource Ecology, Beijing Normal University, Beijing 100875, China; 4 College of Resources Science & Technology, Beijing Normal University, Beijing 100875, China; 5 College of Chemistry and Chemical Engineering, Xinjiang Normal University, Xinjiang 830054, China; 6 School of Chemical Engineering and Technology, Tianjin University, Tianjin 300350, China

**Keywords:** *Peganum harmala* L., NMR spectroscopy, metabonomics

## Abstract

Xerophytes play an active role in preventing soil denudation and desertification in arid and semi-arid areas. *Peganum harmala* L. (Zygophyllaceae family), a seasonally growing, poisonous and drought-tolerant plant, is widely distributed in the Xinjiang Uygur Autonomous Region and used as a traditional herbal medicine as well as, in winter, a fodder source. Previous research has focused on the pharmacological activity of isolated compounds and stress responses to growth environments. However, the metabolic profile of *P. harmala* and variations in its metabolites, including medicinally active and stress resistance components, have not been illustrated during different growth stages. Here, we collected plant samples in May, August, October and December. We determined the metabolic composition of methanol extracts using NMR spectroscopy, and comparisons of four growth stages were accomplished by applying statistical analysis. The results showed that vasicine, choline and sucrose were significantly elevated in samples harvested in May. Significantly higher amounts of betaine, lysine, 4-hydroxyisoleucine and proline were found in samples collected in August than in samples collected in other months, and the concentrations of phosphorylcholine, glucose, acetic acid and vasicinone were highest in December. The relationships between differential biomarkers and plant physiological states affected by diverse growth environmental factors were discussed. Our result deepened the understanding of metabolic mechanisms in plant development and confirmed the advantage of using NMR-based metabolomic treatments in quality evaluation when *P. harmala* is used for different purposes.

## 1. Introduction

*Peganum harmala* L. (*P. harmala*), a member of the Zygophyllaceae family, is a typical xerophyte and halophyte plant that may grow to 30–100 cm high. The most common habitats of this plant are semi-arid rangeland, steppe areas and sandy soils [1]. This vegetation is widely distributed in the Xinjiang Uygur Autonomous Region in the northwest area of China, which covers approximately 2 million hectares [2]. As a drought-resistant and salt-tolerant species [1], *P. harmala* plays a crucial bionomic role in plant defence against disease and insects [3], water and soil conservation, the maintenance of biological diversity, and ecosystem restoration [4].

*Peganum harmala* L. is considered a common nutritious forage grass in winter due to its high crude protein and crude fat content [5] as well as its remarkable ability to adapt to harsh ecological environments and has also shown strong competitiveness in the degradation of grassland [6]. However, in dry years, domesticated animals, especially young individuals, are susceptible to poisoning by *P. harmala*, and the plant also acts as an abortifacient when digested by pregnant animals [7–9]. Previous studies demonstrated that pharmacologically active alkaloids, including β-carboline and quinazoline derivatives, are responsible for this phenomenon [10]. In addition, the aboveground part of *P. harmala* has been used as a local traditional herbal medicine for hundreds of years in China for treating hypertension, cough, diabetes and other conditions [11], and the plant is also used traditionally as an abortifacient and emmenagogue agent in the Middle East and North Africa [5]. A series of studies showed that *P. harmala* is a rich source of medicinal bioactive metabolites with high biological and chemical diversity [12].

In consideration of the potential medicinal and commercial values of this wild pharmaceutical plant, studies on the target metabolites of *P. harmala* have increased with an emphasis on its pharmacological and therapeutic effects and stress response mechanism. Harmine increases human pancreatic beta cell replication by interaction with dual specificity tyrosine-regulated kinase-1a [13]. The antiproliferative activity of alkaloids (vasicinone, harmine, and harmalacidine) isolated from *P. harmala* was confirmed by Lamchouri *et al*. by evaluating their cytotoxic effects on the Jurkat clone E6-1 cell line [14]. Additionally, the extract of *P. harmala* also showed multiple bioactivities such as antibacterial [15], antioxidation and anti-inflammatory capacities [12]. Meanwhile, the response and tolerance of *P. harmala* induced by different environmental stress factors such as drought [16], high salinity [17] and inorganic ions [18] were investigated. Since plants contain multiple chemical constituents, including alkaloids, steroids, flavonoids, anthraquinones, amino acids and inorganic elements [19] and the chemical composition of *P. harmala* varies according to climate conditions, growth years, geographical distribution and sample collection time, finding an effective tool to monitor the dynamic variations in metabolite composition during plant development is crucially important to enhance bioavailability.

Ecological metabolomics are an effective and promising approach for elucidating ecological topics in multiple areas, including stress response, life-history variation, species lifestyle, population structure, nutrient cycles and trophic relationships [20]. Metabolic pathways and other biological processes are regulated by plant development as well as other influential factors from the environment, resulting in fluctuations at the metabolic level [21,22]. As a burgeoning methodology, metabolomics addresses the concomitant linkage between latent metabolic biomarkers and causative physiological mechanisms through a multivariate analysis of complex datasets [23,24]. Nuclear magnetic resonance (NMR), which can simultaneously measure all proton-containing metabolites, is one of the most commonly used methods for metabolite detection in plant science [25]. NMR techniques combined with chemometric techniques have successfully revealed metabolic variations accompanying animal and plant growth [26]. Currently, although this xerophyte and halophyte plant has shown high medicinal and commercial value, there is no metabolomics-based systematic investigation of the metabolome of *P. harmala*.

In this study, dynamic metabolic changes during the growth of wild *P. harmala* (Zygophyllaceae) were characterized using ^1^H NMR spectroscopy combined with multivariate chemometrics. We depicted the metabolic profiles of *P. harmala* using methanol extracts and demonstrated the specificity caused by four different growth stages. The result showed that a metabolomic approach is a powerful tool to accurately discriminate growth stages and that several key biomarkers can be easily quantified to aid in depicting molecular features. This investigation provides data that can be used in the quality control of this medically and commercially significant wild plant and serve as a basis for future *P. harmala* chemical ecology studies.

## 2. Material and methods

### 2.1. Plant material and environmental conditions

The sample collection was conducted in Liuhuanggou Town (86°2′–86°48′ E, 46°39′–47°24′ N) in the Changji Hui Tribe Autonomous Prefecture, Xinjiang Province, northwestern China. This region is located at the northern slope of the Tianshan Mountains and the southern edge of Junggar basin, with an altitude of 1013–1276 m.a.s.l. Under the typical continental-arid climate, the annual natural precipitation of Changji was 316.2 mm in 2014. The mean annual air temperature is 7.74°C, with the lowest monthly mean temperature being −13.08°C (in January) and the highest being 24.18°C (in July). The data showed that this area received abundant sunshine, with an annual duration reaching 2572 h. Climate data of four harvest months (i.e. daily values of mean air temperature, mean relative humidity, duration of sunshine and rainfall) during the field trial are given as supplementary materials (electronic supplementary material, table S1).

The details of plot design for sampling are described in electronic supplementary material, File S1. Six adult *P. harmala* individuals of approximately 50 cm in diameter were harvested in the same field once per season in 2014 (i.e. in May, August, October and December). A total of 24 samples were air-dried, ground to powder and then mixed thoroughly. Due to the loss of moisture in plant tissue readily occurring in a short time, metabolite decomposition caused by enzyme-mediated reactions is well inhibited. Grinding experiments were carried by using an electric grinder (Beijing Junhao Science and Technology Development Co. Ltd, China). All coarse particles in sieving step were ground again until all sample powder passed through the sieve, preventing bias.

### 2.2. Polar metabolite extraction and sample preparation for NMR

First, 2.0** **g of dried sample powder was transferred into a 50 ml centrifuge tube, and 12 ml of methanol (AR, Beijing Chemical Works, China) was added to the tube. The mixture was vortexed (Thermomixer comfort, Eppendorf, Hamburg, Germany) for 30** **s at room temperature and then sonicated (ultrasonic cleaners, Kunshan Ultrasonic instruments Co. Ltd) for 20** **min without heating. This procedure was repeated three times for each sample for complete extraction, and supernatant was then collected and evaporated to dryness under reduced pressure (vacuum rotatory evaporator, Shanghai Yron Chemical Instrument Factory, China). Then, 2 ml of water was added to the methanol extracts, and samples were lyophilized (vacuum freeze drier, ZIRBUS, Germany) and maintained at −80°C. For each growth stage, five collected samples were used for analysis.

Before NMR analysis, the polar extracts (10** **mg) were redissolved in 300 µl of phosphate-buffered saline (pH 7.4), and 200 µl of deuterated water (D_2_O, 99.9%, Cambridge Isotope Laboratories, Andover, MA) solution containing 2** **mM sodium 3-trimethylsilyl [2, 2, 3,3-D_4_] propionate (TSP, 99.0%, Bailingwei Technology Co. Ltd) was added as an intensity reference for a field-frequency lock in the ^1^H NMR experiments. In addition, 200 µl of sodium azide (AR, Beijing Chemical Works, China) solution (10** **mM H_2_O) was added to prevent bacterial growth. A total of 700 µl of solution was vortexed and then transferred to 2 ml Eppendorf centrifuge tubes. The samples were centrifuged at 16 000** **rpm for 20 min, and 600 µl of supernatant was transferred into NMR tubes.

To confirm resonance assignments for marker metabolites, silica gel column chromatography was conducted on the methanol crude extracts with a gradient elution using a mixture of methanol and chloroform. Fractions were repeatedly pooled and purified according to thin-layer chromatography profiles and ^1^H NMR spectra until pure isolated compounds were obtained.

### 2.3. NMR data acquisition

All spectra were acquired on a Bruker DRX-500 spectrometer (Bruker BioSpin GmbH, Rheinstetten, Germany) equipped with a 5** **mm BBFO probe at 298** **K and operating at a proton frequency of 500.13** **MHz. Solvent suppression was accomplished by using a presaturation method with a standard noesypr1d pulse sequence. In total, 256 transients were obtained with 32 k data points, covering a spectral width of 6001.56** **Hz with a relaxation delay of 2.0** **s. A line-width broadening factor of 0.3** **Hz was applied to the free induction decay (FID) before Fourier transformation. Two-dimensional total correlation spectroscopy (^1^H-^1^H TOCSY), homonuclear correlation spectroscopy (^1^H-^1^H COSY), heteronuclear single quantum coherence (^1^H-^13^C HSQC), heteronuclear multiple-bond correlation (^1^H-^13^C HMBC) and 2D *J*-resolved experiments were acquired as auxiliary resonance assignment methods. To obtain clean 2D TOCSY spectra, a data matrix of 256 × 2048 points with 24 scans for each increment and a relaxation delay of 2** **s was used. For the COSY spectra, a data matrix of 400 × 4096 points with 24 scans for each increment and a relaxation delay of 2** **s was used. For the HSQC spectra, a data matrix of 256 × 1024 points covering 20 831 × 3703** **Hz with 24 scans for each increment and a relaxation delay of 2** **s was used. For the HMBC spectra, a data matrix of 128 × 4096 points covering 27 932 × 3703** **Hz with 48 scans for each increment and a relaxation delay of 1** **s was used. Finally, 2D *J*-resolved spectra with presaturation were recorded with a data matrix of 78 × 5000 points covering 8192 × 40** **Hz with 96 scans for each increment.

### 2.4. NMR data preparation and multivariate statistical analysis

All ^1^H NMR spectra were phased and then baseline corrected using processing software (MestReNova, v. 8.1.2). The obtained spectra were calibrated according to an internal standard, and the solvent (water) region from *δ* 4.70–5.05 was excluded from the spectra. For the residual regions (*δ* 0.5–10), binning was performed by adopting a variable bin method named ‘OBA’ (optimized bucketing algorithm), using a 0.02** **ppm bin size and 50% width slackness [1].The buckets were normalized to the whole spectral area and then generated in ASCII format. The resulting files were imported into analysis software (Matlab, R2010a version; Mathworks Inc.). Mean-centring was applied as a data pretreatment method before principal component analysis (PCA) and partial least-squares discriminant analysis (PLS-DA) were performed. PCA was carried out to evaluate the data distribution and determine whether outliers existed using the PLS Toolbox (Eigenvector Research Inc., WA, USA). PLS-DA models were constructed to discriminate the *P. harmala* samples according to their different growth stages, and the resulting models were cross-validated through the leave-one-out method and permutation test. The model performance was estimated by the captured variable R2Y and cross-validated correlation coefficient Q2, whose values are acceptable when larger than 0.5.

Relative quantification based on the internal standard was carried out using the non-overlapped resonances of identified metabolites in ^1^H NMR spectra of polar extracts. The resulting data are shown as the means ± standard deviation (s.d.). The significant differences in metabolite concentrations among the four groups were analysed with a one-way analysis of variance (ANOVA), followed by a post hoc Student–Newman–Keuls (S-N-K) analysis; *p*-values less than 0.05 were considered to indicate statistically significant differences. Heat map and correlation analyses were both used to obtain a comprehensive understanding of the trend in metabolite variation.

## 3. Results

### 3.1. Metabolite identification and structure elucidation

**Figure 1.**
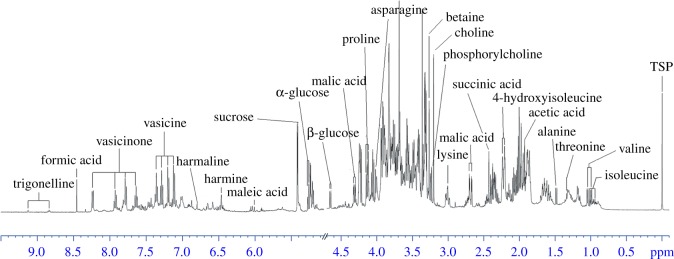
Typical 500** **MHz ^1^H NMR spectra of methanol extracts from *P. harmala*. Annotations for identified metabolites are displayed (see electronic supplementary material, table S2 for assignment).

The metabolic profiles of methanol extracts from *P. harmala* plants collected in December are shown in figure 1, and the structure and assignment of metabolites are listed in electronic supplementary material, table S2. The metabolite resonances from several representative samples were identified by comparison with the literature data [27,28], the SpinAssign database [29], the SpinCouple database and the Human Metabolome Database (HMDB) [30,31]. These assignments were further verified through 2D NMR data (COSY, TOCSY, HSQC, HMBC and *J*-resolved spectra) and ^13^C data from isolated pure compounds (electronic supplementary material, figure S1). Resonances in the spectral region from *δ* 3.2–5.9 were mainly attributed to carbohydrates. The characteristic signals at *δ* 5.24 (d, *J* = 3.8** **Hz) and *δ* 4.65 (d, *J* = 8.2** **Hz) were the anomeric proton resonances of α-glucose and β-glucose, respectively. Sucrose was identified and confirmed by an inspection of the cross peaks in the HSQC spectrum. The doublet at *δ* 5.42 (d, *J* = 3.8** **Hz), which correlates to multiplets at *δ* 3.69, *δ* 3.56, *δ* 3.78 and *δ* 3.49 in the COSY spectrum, represents the glucose unit of sucrose. Signal peaks of choline, phosphorylcholine, betaine, proline and 4-hydroxyisoleucine were clearly visible in midfield spectral region. For example, the characteristic singlet at *δ* 3.27 was identified as the N^+^(CH_3_)_3_ group from betaine, and the cross peak in the HSQC spectrum indicated that the signal correlated with the methyl carbon at *δ* 53.39. Proline was identified through the doublet (dd, *J* = 8.63, 6.56** **Hz) corresponding to the H*α* carboxylic acid at *δ* 4.12 (bonded to a carbon atom at *δ* 62.0), which was correlated via COSY to two multiplets corresponding to the protons of the methylene group at *δ* 2.06 and *δ* 2.35. 4-Hydroxyisoleucine was identified through multiple peaks corresponding to the H*α* carboxylic acid at *δ* 4.25 (bonded to a carbon atom at *δ* 62.2). The resonances at *δ* 4.25, *δ* 3.91, *δ* 3.32, *δ* 2.23 and *δ* 1.80 were correlated in one spin system via TOCSY, and the corresponding carbon resonances at *δ* 62.2, *δ* 53.8, *δ* 38.5, *δ* 32.9 and *δ* 28.2** **ppm were assigned via HSQC.

Four species-specific alkaloids (vasicine, vasicinone, harmine and harmaline) were isolated from methanol extracts and identified with the assistance of correlation between resonances in the aromatic region of the NMR spectra and the ^13^C data of pure compounds. Electronic supplementary material, figure S2A shows the aromatic region with assignments of spin systems indicated by arrows. Electronic supplementary material, figure S2B and S2C depicts the expanded portion of 2D TOCSY and HSQC spectra, respectively [32]. Spin systems from vasicine and vasicinone were recognized via the TOCSY spectra. The cross peaks indicated that the proton signals at *δ* 7.65, *δ* 7.79, *δ* 7.94 and *δ* 8.25 (corresponding carbon resonances: *δ* 127.54, *δ* 126.27, *δ* 134.67 and *δ* 126.84) were in the same spin system and that the proton signals at *δ* 7.12, *δ* 7.20, *δ* 7.28 and *δ* 7.36 (corresponding carbon resonances: *δ* 116.9, *δ* 126.02, *δ* 127.4 and *δ* 129) were in another spin system. Electronic supplementary material, figure S2C shows the direct ^1^H/^13^C correlations for a-5, a-6, a-7 and a-8; b-5, b-6, b-7 and b-8; and c-1 and c-2 (atom labels shown in electronic supplementary material, figure S3).

The levels of 13 metabolites were determined using the integration values of characteristic signals with respect to the intensity of internal standard TSP. The types of compounds that were observed included alkaloids (vasicine and vasicinone), organic acids (acetic acid and malic acid), sugars (α-glucose, β-glucose and sucrose), amino acids (4-hydroxyisoleucine, lysine, betaine and proline), and essential compounds in cell membrane biosynthesis (choline and phosphorylcholine). The concentrations of these metabolites during four growth periods are shown in electronic supplementary material, table S3.

### 3.2. Statistical analysis of ^1^H NMR data

**Figure 2.**
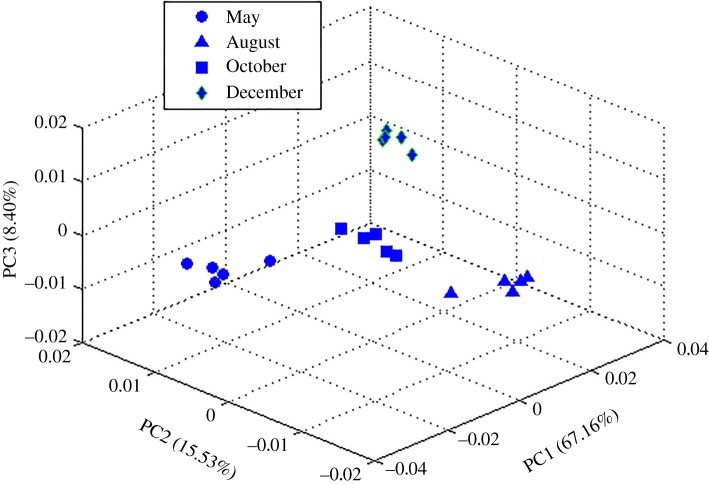
PCA scores plots for the methanol extracts of *P. harmala* samples harvested in May (filled circle), August (filled triangle), October (filled square) and December (filled diamond).

**Figure 3.**
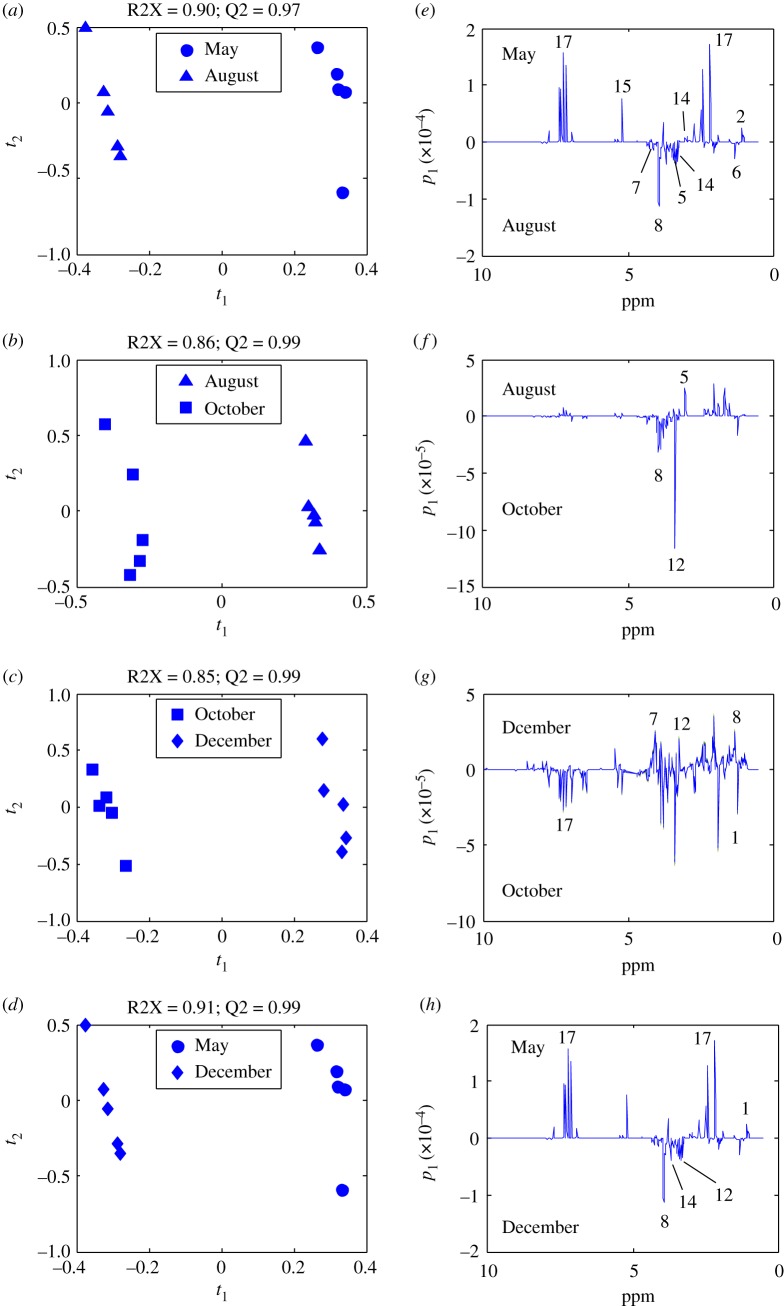
PLS-DA score plots and linear loading diagrams. PLS-DA score (*a*–*d*) and loading (*e*–*h*) plots for the methanol extracts of *P. harmala* harvested in May (filled circle), August (filled triangle), October (filled square) and December (filled diamond). Score plots show the satisfactory separation between subgroups.

Data from four adjacent season pairs (spring–summer, summer–autumn, autumn–winter, winter–spring) were investigated. PCA was a useful tool for extracting and displaying the systematic variation in a data matrix to identify trends and clusters in an unsupervised manner. From the score plot, four clearly separated clusters were identified by the PCA without any notable outliers; 91.1% of the total variance was explained by three principal components (PCs) (figure 2), with PC1 accounting for 67.2% of the total variance, PC2 accounting for 15.5%, and PC3 accounting for 8.3%. Although the PCA models have adequately summarized the information of the dataset and revealed intergroup differences caused by physiological state variations, supervised PLS-DA analysis was still conducted for the purpose of strengthening the differences, filtering noise more efficiently and depicting the potential biomarkers more accurately. Pairwise comparisons between these four growth stages, as shown in figure 3, were carried out, and metabolic variations were detailed.

PLS-DA score plots maximized differences between subgroups with satisfactory quality parameters (A: R2X = 0.89, Q2 = 0.98; B: R2X = 0.88, Q2 = 0.98; C: R2X = 0.79, Q2 = 0.99; D: R2X = 0.94, Q2 = 0.98), indicating the robustness of the statistical models. In addition, permutation tests showed good predictability (electronic supplementary material, figure S3). Figure 3*e*–*h* shows loading plots along with assignment of the major differential metabolites.

From the above multivariate analysis, relevant physiological information was acquired, and the variables responsible for the group separation were identified from the loading plots of these four pairs of months. The loading plot of the May–August group indicated that the levels of vasicine (17), sucrose (15), choline (12) and valine (2) are higher in May samples than August samples and that lysine (5), acetic acid (6), proline (7), 4-hydroxyisoleucine (8) and betaine (14) levels were lower in the May samples (figure 3*e*). The loading plot of the August–October groups revealed that the separation was dominated by high concentrations of 4-hydroxyisoleucine (8) and phosphorylcholine (13) in the October samples and that higher levels of lysine (5) were observed in the August samples than the October samples (figure 3*f*). Higher levels of proline (7), 4-hydroxyisoleucine (8) and choline (12) were found in the December group than in the October group (figure 3*g*). For the last group, the contents of vasicine (17), isoleucine (1) in the May group were higher than those in the samples of December, and 4-hydroxyisoleucine (8), choline (12), betaine (14) levels were higher in December samples than May samples (figure 3*h*).

### 3.3. Relative quantification of identified metabolites and correlation analysis

**Figure 4.**
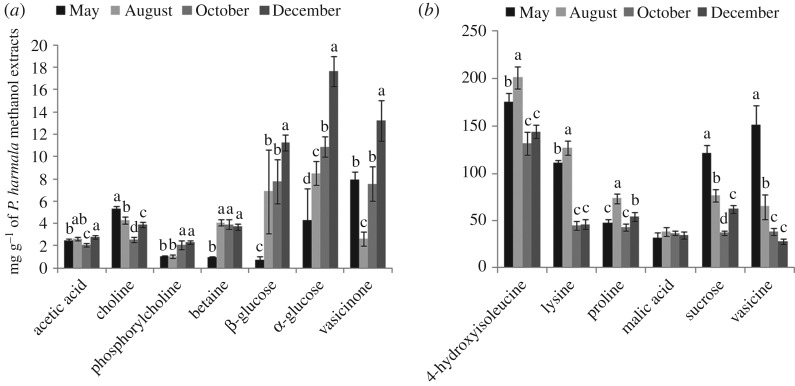
Histogram of concentrations of methanol metabolites of *P. harmala* calculated from NMR signals. Concentrations are given in mg g^−1^, and data are shown as the mean concentrations (*n* = 5) with an error bar. (*a*) The contents of metabolites are in the 0–20 mg g^−1^ range and (*b*) in the 0–250 mg g^−1^ range. Note: Data are given as the mean ± s.d. (standard deviation). Different letters within same group represent significant differences at the 0.05 level, and labels with the same letter are not significantly different at the level of 0.05, as determined by the S-N-K test.

To further investigate the impact of physiological state on metabolic changes, advanced illustration tools were used on the basis of the relative quantification of 13 metabolites (figure 4). Electronic supplementary material, table S3 shows the contents of polar metabolites derived from the samples collected during the four development periods, and *p*-values are shown to indicate significant differences between them. Figure 4 illustrates the concentration variation in the important polar metabolites in the form of histogram.

The samples collected in May from the Xinjiang Changji areas contained more vasicine than samples taken in the other three growth stages. In August, the levels of amino acids in the stems and leaves reached their maximum values, whereas the betaine content of *P. harmala* was not significantly different among the other stages of growth; lysine and 4-hydroxyisoleucine in the extracts exhibit the same trend as plant development. The contents of choline and sucrose had a tendency to decrease from May to October and began to accumulate in December. The content of α-glucose, β-glucose, vasicinone, phosphorylcholine and acetic acid reached peak values in December.

**Figure 5.**
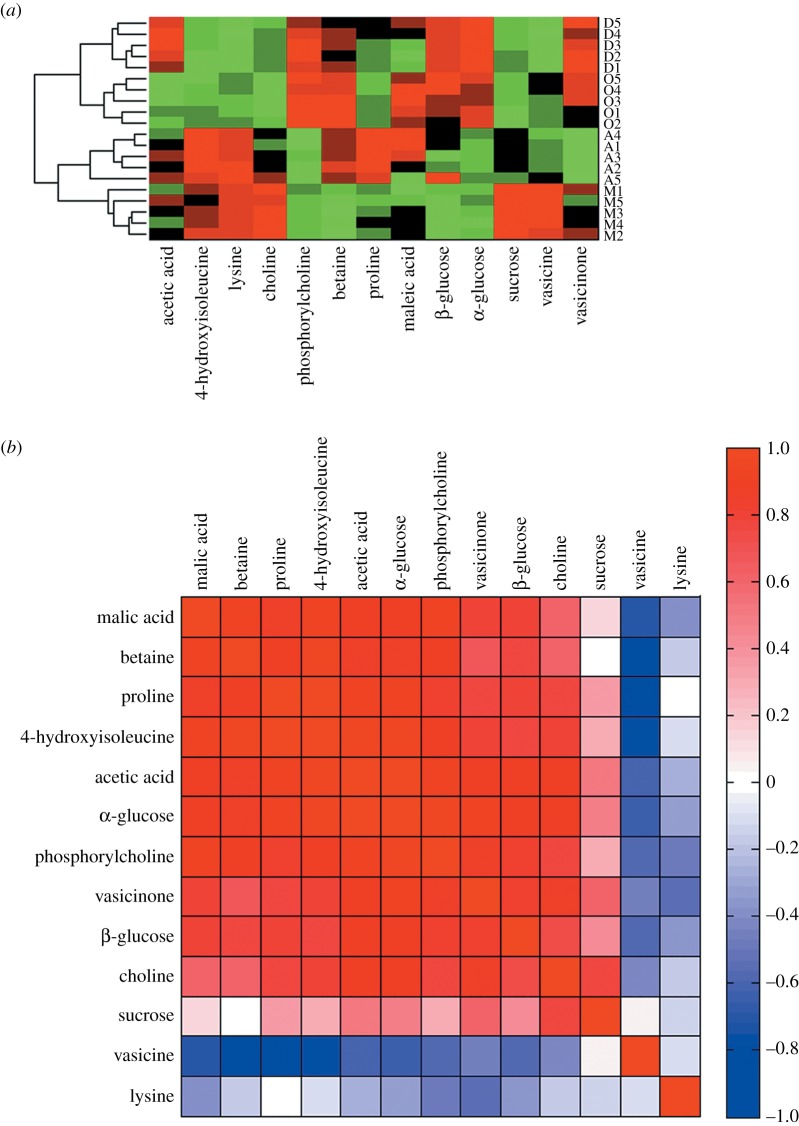
Heat map and correlation analysis. Heat map (*a*) of identified metabolites from four months (M: May; A: August; O: October; D: December; positive correlation (red) or negative correlation (green). Rows: samples; columns: metabolites). The hierarchical clustering analysis used a Euclidean distance metric and average linkages to generate the hierarchical tree. Correlation analysis (*b*) of the differential metabolites for four growth periods. It is a pseudocolour map corresponding to the correlation matrix returned by Pearson correlation analysis. The colours in (*b*) represent values of Pearson's correlation coefficient (positive correlation (red) or negative correlation (blue)).

Heat maps and correlation maps were introduced to analyse the correlations between the metabolites and growth stages and among the metabolites themselves. Through heat map analysis (figure 5*a*), we found that the 20 samples from the four growth stages are well clustered. The heat map showed that collecting samples in May was clearly positively correlated with vasicine, sucrose, choline, lysine and 4-hydroxyisoleucine levels but also significantly negatively correlated with phosphorylcholine, betaine, glucose and proline levels. Collecting samples in the summer was positively correlated with lysine, 4-hydroxyisoleucine, betaine, proline and malic acid but negatively correlated with phosphorylcholine, glucose, vasicine and vasicinone. The metabolic changes during October and December were similar. The content of α-glucose, β-glucose, betaine, phosphorylcholine and vasicinone showed positive relationships. The vasicine, sucrose, choline, lysine and 4-hydroxyisoleucine content have significant negative correlations with collecting the samples in October. The differential metabolites acetic acid and vasicine displayed a significant positive correlation, whereas malic acid showed a significant negative correlation with collection of the samples in winter. The changes in the concentrations of metabolites reflected the plant adaptation to different climate conditions and its development, and might be related to the complex metabolic network and pathways of *P. harmala*.

In the correlation map, variable reordering was accomplished by using the k-nearest neighbour (KNN) method (figure 5*b*). The correlation between variables is considered to be strong when the absolute value of the correlation coefficient is close to 133. Through cluster analysis, metabolites can be identified as potential biomarkers that may interact in biochemical processes. As indicated, 4-hydroxyisoleucine, lysine, choline, sucrose and vasicine are highly positively correlated with one another and show weak positive relationships with proline and acetic acid in addition to negative relationships with the rest of the identified metabolites.

**Figure 6.**
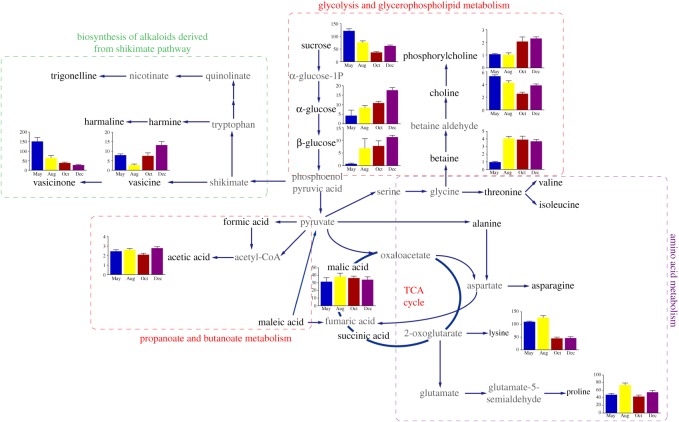
Schematic metabolic pathway network analysis of *P. harmala* responses to different growth stages based on the findings and information obtained from the KEGG database. Metabolite changes are represented in bar graphs. Metabolites coloured grey are not detected. Single solid arrows represent one-step process, while two connected solid arrows represent multi-steps process. Dash line boxes with different colour indicate metabolism networks of particular metabolites.

To facilitate the visualization of these results, metabolite networks for the identified metabolites and their differences were elucidated in figure 6 based on the quantitative data and information obtained from the KEGG database.

## 4. Discussion

Plants interact with various environmental stress factors, including herbivory by animals and insects, pathogens, metals, and ultraviolet radiation. All these stress factors activate a series of defence mechanisms and then qualitatively and/or quantitatively influence plant metabolomes [27]. *Peganum harmala* L*.* is a perennial herb living in a harsh environment that includes an arid climate, low precipitation, relatively high evaporation, long periods of sunshine and large temperature differences between day and night, with seasonal periodicity in plant growth and development [28].

In the arid spring, the aboveground part of *P. harmala* accumulates carbohydrates and amino acids for biosynthesis. Sucrose, as the product of photosynthesis and a signal molecule in assimilate partitioning, reached its maximum concentration in May, and high sucrose content can be attributed to higher biomass production during vegetative growth [29]. A lower level of sucrose was observed in samples from October, which may be related to the considerable energy required by sucrose catabolism during the seed-forming period. Carbohydrate changes occur in many plant species under cold stress in winter. The highest content of glucose was found in samples from December and thus considered to function as stored energy during the long dormancy period.

Alkaloids, acting as plant regulators, play several important physiological roles in controlling seed germination, allelopathy, UV-B protection, plant-bacterium interactions, antioxidant activity and environmental stress [20]. Plant can also release allelochemicals to inhibit surrounding plant growth and defend against animal feeding to obtain an advantage in ecological population competition. The Zygophyllaceae family is well known to be a natural resource of two important environmentally influenced metabolites: β-carboline and quinazoline alkaloids. Previous results have shown that both β-carboline and the quinazoline alkaloids of *P. harmala* have insecticidal [30] and fungicidal properties and inhibit seed germination [31]. A study has shown that alkaloids delay locust sexual maturity and lead to a reduction in female fecundity, thus reducing the hatching rate of locust eggs [32]. Vasicine is produced from proline and the glutamic semialdehyde biosynthesis of secondary metabolites. Vasicinone is an auto-oxidation product of vasicine, and the major metabolic pathway of this compound involves the shikimate pathway [33]. Vasicine and vasicinone exhibited their maximum concentrations in May, and their high content might be a method that chemical defences use to prevent feeding by herbivores [34]. Since vasicine can be used in the treatment of Alzheimer's disease [35], this result provided valuable information for the quality assessment of this traditional herbal medicine. *Peganum harmala* L. is a nutrient-rich high-quality feed containing abundant sugars, amino acids [5], minerals [36] and flavonoids [37] after the autumn, at which time it also exhibits reduced levels of alkaloids.

In plants, primary metabolites such as amino acids are essential for growth, development, respiration and photosynthesis. 4-hydroxyisoleucine is a plant-derived nonprotein amino acid with a particular physiology and chemical activity that affects glucose and lipid metabolism in this plant [38]. Abundant 4-hydroxyisoleucine was found in *P. harmala* samples, and its content increased from May to August and decreased in October; there were no significant changes between samples from October and December. The abovementioned phenomenon was considered to be affected by the rate of photosynthesis, which correlated with sunshine duration (May: 282.1 h, August: 253.1 h, October: 222.5 h and December: 164.4 h). A similar variation trend was found in the lysine level in the stems and leaves of *P. harmala*.

*Peganum harmala* L*.* possesses a high content of betaine in its last three growth stages. As a quaternary ammonium compound that has non-toxic osmoprotectant properties, betaine could help *P. harmala* improve its salt and drought resistance in the Xinjiang region [39] and serve as a cryoprotectant [40]. It is also involved in the prevention of various abiotic stresses by stabilizing the quaternary structure of proteins [41].

Higher levels of acetic acid and phosphorylcholine were found in samples from December than in samples from the other months. As a signal molecule, acetic acid can act as a germination inhibitor. The occurrence of the bud dormancy of *P. harmala* in winter may be perceived as biological adaptability to the harsh environment during the cold weather (average temperature of −13.08°C). A high content of phosphorylcholine is helpful in promoting biomembrane synthesis, and biomembranes may help plants resist cold weather and maintain normal biological metabolic function during dormancy [42].

## 5. Conclusion

*Peganum harmala* L. is commonly used as forage grass and has numerous pharmacological activities. In our study, ^1^H NMR profiles of methanol extracts were demonstrated, and 24 compounds were identified, including species-specific alkaloids. Multivariate statistical analysis was carried out to discriminate plant growth stages, and several key biomarkers were quantified to assist in depicting metabolic features. The dynamic variations in metabolic profiles and their ecological significance were further discussed in detail. Based on these investigated endogenous metabolite profiles, our preliminary finding suggested that ^1^H NMR-based metabolomic method is an excellent and efficient holistic method for illustrating the physiological states of this xerophyte and halophyte plant. These results provide useful information in the drug exploitation and quality control and sets the stage for more in-depth investigation on this traditional pharmaceutical herb.

## Supplementary Material

Supplementary figures and tables from "NMR-Based Metabolomic Profiling Of Peganum Harmala L. Reveals Dynamic Variations Derived From Different Growth Stages"

## Supplementary Material

Raw data ( 1H NMR specra) obtained from polar metabolite extraction of Peganum harmala L.

## Data accessibility

The datasets supporting this article have been uploaded as part of the electronic supplementary material.
