# Supplementary material for: NMR-based metabolomic profiling of Peganum harmala L. reveals dynamic variations between different growth stages
Source: R Soc Open Sci. 2018 Jul 18;5(7):171722. doi: 10.1098/rsos.171722 (PMC6083650; doi:10.1098/rsos.171722)
Supplement: Supplementary figures and tables from "NMR-Based Metabolomic Profiling Of Peganum Harmala L. Reveals Dynamic Variations Derived From Different Growth Stages" [file rsos171722supp1.docx]

**Electronic supplementary File S1**

Supplemental file S1. Plot design for sampling

Sampling was conducted in the same region in May (spring), August (summer), Octorber (autumn), and December (winter) in 2014, respectively. A square flat ground covered an area of 100×100 m^2^ with distribution of *P. harmala* L．was selected as the sample plot in Liuhuanggou Town in Changji Hui Tribe Autonomous Prefecture. Along the diagonal line of this region, six equidistant quadrats (1×1m^2^) were choosed and the aboveground parts of an individual plant were collected in one small quadrat randomly. These samples should be at a consistent growth stage and the stem of plants are about 50 cm in diameter. The healthy plants without diseases, insect attack and mechanical damage were guaranteed by carefully inspection.

◆

◆

◆

◆

◆

◆

100 m

1 00m

quadrat （1 × 1m^2^）

Sample plot

**Electronic supplementary Table**

Supplemental Table S1．Xinjiang changji region climate data in 2014

| Date | Daily values of mean air temperature  (℃) | Mean relative humidity  (%) | Daily values  rainfall  (mm) | Duration of  sunshine  (h) |
| --- | --- | --- | --- | --- |
| 2014/05/1 | -1.3 | 42 | 0 | 12.2 |
| 2014/05/2 | 1.4 | 37 | 0 | 12.2 |
| 2014/05/3 | 4.3 | 33 | 0 | 10.1 |
| 2014/05/4 | 7.1 | 34 | 0 | 12.2 |
| 2014/05/5 | 7.7 | 27 | 0 | 12.1 |
| 2014/05/6 | 8.6 | 26 | 0 | 11.7 |
| 2014/05/7 | 8.4 | 42 | 0 | 8.4 |
| 2014/05/8 | 3.8 | 34 | 0 | 7.1 |
| 2014/05/9 | -0.7 | 40 | 11 | 8.7 |
| 2014/05/10 | 1.4 | 25 | 0 | 10.3 |
| 2014/05/11 | 0.9 | 38 | 0 | 10.8 |
| 2014/05/12 | 1 | 42 | 0 | 2 |
| 2014/05/13 | -1.6 | 34 | 0 | 12.3 |
| 2014/05/14 | 2.5 | 33 | 0 | 10.3 |
| 2014/05/15 | 5 | 44 | 6 | 6.5 |
| 2014/05/16 | 3.1 | 53 | 4 | 7.7 |
| 2014/05/17 | 1.4 | 39 | 0 | 11.6 |
| 2014/05/18 | 4.1 | 24 | 0 | 12.6 |
| 2014/05/19 | 6.8 | 26 | 0 | 12.3 |
| 2014/05/20 | 10.1 | 29 | <0.1 | 7.7 |
| 2014/05/21 | 3 | 72 | 105 | 2.1 |
| 2014/05/22 | 0.1 | 78 | 80 | 0 |
| 2014/05/23 | -0.9 | 79 | 116 | 1.9 |
| 2014/05/24 | 0.2 | 55 | 4 | 13 |
| 2014/05/25 | 5.9 | 33 | 0 | 13.1 |
| 2014/05/26 | 8.3 | 34 | 0 | 13.1 |
| 2014/05/27 | 8.5 | 35 | 0 | 11.2 |
| 2014/05/28 | 9.4 | 55 | 14 | 6.2 |
| 2014/05/29 | 9.9 | 37 | 0 | 13.1 |
| 2014/05/30 | 12 | 41 | 0 | 9.6 |
| 2014/05/31 | 3.1 | 82 | 121 | 0 |
| 2014/08/1 | 4.6 | 55 | 0 | 10.3 |
| 2014/08/2 | 5.7 | 63 | 0 | 5.4 |
| 2014/08/3 | 4.8 | 43 | 0 | 11.9 |
| 2014/08/4 | 8.8 | 38 | 0 | 9.7 |
| 2014/08/5 | 12.8 | 48 | 12 | 7.2 |
| 2014/08/6 | 9.7 | 56 | 48 | 10.5 |
| 2014/08/7 | 11.9 | 39 | 0 | 9.2 |
| 2014/08/8 | 9.7 | 52 | 8 | 8.6 |
| 2014/08/9 | 11.1 | 48 | 0 | 7.4 |
| 2014/08/10 | 7.5 | 64 | 45 | 11.4 |
| 2014/08/11 | 8 | 45 | 0 | 9.6 |
| 2014/08/12 | 11.3 | 27 | 0 | 11.3 |
| 2014/08/13 | 11.7 | 28 | 0 | 11.1 |
| 2014/08/14 | 14.3 | 31 | 0 | 0.4 |
| 2014/08/15 | 12.8 | 31 | 0 | 9.3 |
| 2014/08/16 | 12.8 | 42 | 0 | 10.2 |
| 2014/08/17 | 11.7 | 68 | 0 | 0 |
| 2014/08/18 | 10.6 | 74 | 4 | 0.4 |
| 2014/08/19 | 10.6 | 75 | 105 | 0.4 |
| 2014/08/20 | 9.4 | 71 | 85 | 2.8 |
| 2014/08/21 | 10.1 | 57 | 31 | 7.4 |
| 2014/08/22 | 11.4 | 41 | 0 | 11.7 |
| 2014/08/23 | 12.1 | 37 | 0 | 11.7 |
| 2014/08/24 | 11.4 | 40 | 0 | 11.5 |
| 2014/08/25 | 12.6 | 36 | 4 | 11.3 |
| 2014/08/26 | 15.2 | 35 | 2 | 8.3 |
| 2014/08/27 | 13.1 | 49 | 0 | 0.2 |
| 2014/08/28 | 7.5 | 59 | 0 | 11.5 |
| 2014/08/29 | 7.5 | 52 | 0 | 11 |
| 2014/08/30 | 9.9 | 40 | 0 | 0 |
| 2014/08/31 | 10.3 | 32 | 0 | 11.1 |
| 2014/10/1 | 4.5 | 51 | 0 | 8.3 |
| 2014/10/2 | 4.2 | 80 | 60 | 1 |
| 2014/10/3 | 1.5 | 64 | 2 | 9.9 |
| 2014/10/4 | 4.9 | 50 | 0 | 8 |
| 2014/10/5 | 3 | 68 | 0 | 7.6 |
| 2014/10/6 | 0.5 | 66 | 0 | 8.6 |
| 2014/10/7 | 2.1 | 69 | 2 | 10 |
| 2014/10/8 | 1.6 | 72 | 0 | 6.5 |
| 2014/10/9 | -3.9 | 84 | 131 | 0 |
| 2014/10/10 | -6.3 | 81 | 58 | 5.9 |
| 2014/10/11 | -7.3 | 66 | 1 | 9.2 |
| 2014/10/12 | -3.8 | 66 | 0 | 10.2 |
| 2014/10/13 | -0.7 | 40 | 0 | 9 |
| 2014/10/14 | -2.8 | 49 | 0 | 8.9 |
| 2014/10/15 | 0.1 | 46 | 0 | 8.7 |
| 2014/10/16 | 2.5 | 39 | 0 | 8.7 |
| 2014/10/17 | 1.2 | 44 | 0 | 8.6 |
| 2014/10/18 | 1.5 | 41 | 0 | 8.6 |
| 2014/10/19 | 1.5 | 37 | 0 | 8.9 |
| 2014/10/20 | 4.7 | 29 | 0 | 8.4 |
| 2014/10/21 | 4.5 | 35 | 0 | 7.8 |
| 2014/10/22 | 1.2 | 62 | 0 | 8.3 |
| 2014/10/23 | 2.9 | 42 | 0 | 8.5 |
| 2014/10/24 | 3.2 | 53 | 0 | 4.8 |
| 2014/10/25 | 2.6 | 77 | 0 | 5.5 |
| 2014/10/26 | 3 | 46 | 0 | 8.3 |
| 2014/10/27 | -3.4 | 64 | 26 | 0.6 |
| 2014/10/28 | -7.5 | 86 | 16 | 0 |
| 2014/10/29 | -8.8 | 74 | <0.1 | 7.9 |
| 2014/10/30 | -6.5 | 50 | 0 | 7.9 |
| 2014/10/31 | -8.6 | 66 | 0 | 7.9 |
| 2014/12/1 | -12.7 | 43 | 0 | 5.9 |
| 2014/12/2 | -14.6 | 59 | 0 | 6.6 |
| 2014/12/3 | -10.4 | 44 | 0 | 6.5 |
| 2014/12/4 | -10.1 | 55 | 0 | 5.5 |
| 2014/12/5 | -10.6 | 82 | 12 | 0 |
| 2014/12/6 | -15.3 | 66 | 2 | 6.3 |
| 2014/12/7 | -11.7 | 59 | 0 | 5.3 |
| 2014/12/8 | -16.8 | 78 | 107 | 0 |
| 2014/12/9 | -20.8 | 64 | 4 | 6.2 |
| 2014/12/10 | -20 | 64 | 18 | 2.6 |
| 2014/12/11 | -16.2 | 68 | 11 | 6 |
| 2014/12/12 | -16.3 | 62 | 0 | 6.2 |
| 2014/12/13 | -15.4 | 63 | <0.1 | 0 |
| 2014/12/14 | -21.1 | 68 | 4 | 5.5 |
| 2014/12/15 | -18 | 54 | 0 | 6.2 |
| 2014/12/16 | -16 | 41 | 0 | 6.2 |
| 2014/12/17 | -11.1 | 37 | 0 | 5.5 |
| 2014/12/18 | -10.3 | 46 | 0 | 6.2 |
| 2014/12/19 | -10.1 | 60 | 0 | 6.1 |
| 2014/12/20 | -9.3 | 64 | 0 | 5.8 |
| 2014/12/21 | -11.6 | 61 | 0 | 6.2 |
| 2014/12/22 | -8.1 | 53 | 0 | 5.7 |
| 2014/12/23 | -10.3 | 58 | 0 | 6.2 |
| 2014/12/24 | -10.1 | 57 | 0 | 6.1 |
| 2014/12/25 | -10.1 | 50 | 0 | 5.2 |
| 2014/12/26 | -7.9 | 48 | <0.1 | 5.4 |
| 2014/12/27 | -12 | 44 | <0.1 | 6.2 |
| 2014/12/28 | -6.9 | 38 | 0 | 6.4 |
| 2014/12/29 | -9.1 | 43 | 0 | 5.9 |
| 2014/12/30 | -9.1 | 39 | 0 | 6.2 |
| 2014/12/31 | -7.8 | 41 | 0 | 6.3 |

Supplemental Table S2． Mostly common metabolites found in Peganum harmala L by nuclear magnetic resonance (NMR) analysis.

| number | compound | chemical structure formula | ^1^H ppm | ^13^C ppm | COSY | HMBC |
| --- | --- | --- | --- | --- | --- | --- |
| 1 | isoleucine |  | [1]  [2]3.48 (d, 7.06 Hz)  [6]1.02 (d, 7.06 Hz)  [5]0.95 (t, 7.15 Hz) | 174.3  59.5  14.6  11.1 | H-5/H-6 |  |
| 2 | valine |  | [3]2.22 (m)  [4]1.05 (d, 7.0 Hz)  [5]0.99 (d, 7.0 Hz) | 32.84  17.93  16.58 | H-4/H-5 | H-5/C-3 |
| 3 | threonine |  | [2]3.57 (d, 4.86Hz)  [3]4.25 (m)  [4]1.33 (d, 6.55 Hz) | 61.1  66  19.8 | H-2/H-4  H-2/H-3 |  |
| 4 | alanine |  | [2]3.57 (m)  [3]1.48 (d, 7.3Hz) | 50.4  16.4 | H-2/H-3 |  |
| 5 | lysine |  | [1] 3.6 (m)  [2] 1.65, 1.89 (m)  [3] 2.25 (m)  [4] 3.01, 3.4 (m) * | 59.3  21.9  26.6  43.9 | H-2/H-4  H-2/H-3  H-3/H-4 |  |
| 6 | acetic acid |  | [2]1.92 (s)* | 23.3 |  |  |
| 7 | proline |  | [2] 4.12 (dd, 8.63, 6.56 Hz) *  [3]3.30–3.35 (m),  [4]2.31–2.37 (m)  [5]1.95–2.00 (m)  [6] | 62  43.9  29  23.9  174.3 | H-2/H-3  H-3/H-4  H-4/H-5 |  |
| 8 | 4-hydroxyisoleucine |  | [2]4.25 (m)  [3]3.32 (m)  [4]3.91 (m)  [5]2.22*, 1.95 (m)  [6]1.88 (m) | 62.2  38.5  53.8  32.9  28.2 | H-2/H-6  H-2/H-5  H-3/H-6  H-4/H-6  H-4/H-5 | H-2/C-3  H-2/C-4  H-2/C-6 |
| 9 | [succinic](javascript:void(0);) [acid](javascript:void(0);) |  | [2]2.43 (s)  [3]2.43 (s) | 33.6 |  |  |
| 10 | malic acid |  | [3a]2.67(dd,2.9,15.62 Hz)  [3b]2.39 (dd, 11.44, 15.62 Hz)  [2]4.31 (dd, 3.1, 10.2 Hz) * | 42.46  42.46  70 | H-2/H-3a  H-2/H-3b  H-3a/H-3b |  |
| 11 | asparagine |  | [2]4.03 (dd, 4.4,7.25 Hz)  [3a]2.94 (m)  [3b]2.84 (m) | 51.59  34.53  34.53 | H-2/H-3a  H-2/H-3b  H-3a/H-3b |  |
| 12 | choline |  | [1]4.07  [3]3.21 (s) * | 55.69  53.92 |  |  |
| 13 | phosphorylcholine |  | [3]3.23 (s)* | 54.08 |  |  |
| 14 | betaine |  | [3]3.27 (s) * | 53.39 |  |  |
| 15 | sucrose |  | [1]3.69 (s)  [3]4.22 (d, 8.65 Hz)  [4]4.06 (t, 8.89Hz)  [5]3.91 (m)  [6]3.87 (d, 3.15)  [1’]5.42 (d, 3.8Hz) *  [2’]3.59 (m)  [3’]3.78 (t, 9.48 Hz)  [4’]3.49 (t, 9.28 Hz)  [5’]3.91 (m) | 61.2  76.3  74.1  81.3  92.34  71.2  72.5  69.3 | H-1’/H-2’ | H-1’/C-4 |
| 16 | β-glucose |  | [1]4.65 (d, 8.0Hz)* | 95.9 |  |  |
| 17 | vasicine |  | [1]3.77, 3.69 (m)  [2] 2.15, 2.71 (m)  [3] 5.22 (t, 8.80Hz)  [5] 7.10 (m)  [6] 7.36 (m)  [7] 7.28 (m)*  [8]7.19(d, 7.5Hz)  [9]4.72,4.61 (dd, 8.5,5.0 Hz)  [3a]  [5a]  [8a] | 50.6  29.4  71  117  129  127.4  126  46.5  163.7  130.35  116.61 | H-3/H-5  H-3/H-1  H-3/H-2  H-1/H-2  H-6/H-7 | H-6/C-5  H-6/C-7  H-2/C-1  H-3/C-2 |
| 18 | α-glucose |  | [1] 5.21 (d, 3.83Hz)* |  |  |  |
| 19 | [maleic](javascript:void(0);) [acid](javascript:void(0);) |  | [2,3]6.01 (s) |  | H-2/H-3 |  |
| 20 | harmine |  | [1]6.46 (d, 1.9Hz)  [2]6.69 (dd, 8.75,2.25 Hz)  [4]7.84 (d, 5.64Hz)  [6]2.55 (s)  [8]7.69 (d, 5.42Hz)  [9]7.61 (d, 8.59Hz)  [10]3.84 (s) | 93.4  111  128.1  19.1  113  123  55.6 | H-8/H-1  H-9/H-4  H-2/H-10  H-9/H-6 | H-8/C-1 |
| 21 | harmaline |  | [1]6.64 (dd, 8.85,1.94Hz)  [2]6.83 (d, 1.66Hz)  [4]7.34 (d, 8.64Hz)  [6]2.47 (s)  [8] 2.96 (t, 8.83Hz)  [9] 3.76 (t, 8.83Hz)  [10]3.81 (s) | 94  112.5  123.4  18  18.5  41.7  55.5 | H-4/H-3  H-8/H-9 | H-4/C-1 |
| 22 | vasicinone |  | [1]4.11, 3.91 (m)  [2]1.99, 2.45 (m)  [3]4.99（t，6Hz）  [5]7.71(m)  [6]7.93(m)  [7]7.64(m)  [8]8.25(d, 6 Hz)*  [9]  [3a]  [5a]  [8a] | 43.39  30.02  71.8  126.6  135.4  127.6  125.1  161.39  160.37  149.57  121.07 | H-8/H-6  H-8/H-7  H-6/H-5  H-6/H-7 | H-8/C-6  H-5/C-7  H-2/C-3  H-1/C-3 |
| 23 | formate |  | [1]8.46 (s) |  |  |  |
| 24 | trigonellie |  | [1]9.12（s)  [3]8.83（m) | 148.41  147.51 |  |  |

Multiplicity: s, singlet; d, doublet; t, triplet; q, quartet; dd, doublet of doublets; m, multiplet；*, measured resonance peaks.

Supplemental Table S3**．** Relative quantification (mg/g, with respect to internal standard) of principal metabolites by the integration values of ^1^H NMR resonances (Mean ± std mg/g)

| Metabolite | May | August | October | December | *P* |
| --- | --- | --- | --- | --- | --- |
| Vasicine | 151.4 ± 20.70a | 64.91 ± 12.90b | 38.37 ± 4.16c | 27.48 ± 2.81c | 4.71E-04 |
| Vasicinone | 7.93 ± 0.69b | 2.62 ± 0.65c | 7.56 ± 1.57b | 13.23 ± 1.84a | 0.001 |
| β-Glucose | 0.74 ± 0.27c | 6.87 ± 3.78b | 7.77 ± 2.01b | 11.27 ± 0.70a | 0.0027 |
| α-Glucose | 4.28 ± 2.87d | 8.48 ± 1.06c | 10.88 ± 0.93b | 17.63 ± 1.34a | 6.95E-04 |
| Sucrose | 121.64 ± 8.46a | 76.46 ± 5.89b | 36.81 ± 2.62d | 62.42 ± 3.55c | 4.68E-04 |
| 4-Hydroxyisoleucine | 176.70 ± 9.04b | 201.78 ± 11.02a | 132.19 ± 12.69c | 144.42 ± 7.02c | 9.31E-04 |
| Lysine | 110.69 ± 2.48b | 126.98 ± 7.81a | 45.04 ± 5.15c | 46.41 ± 5.12c | 0.0011 |
| Betaine | 0.96 ± 0.11b | 4.07 ± 0.26a | 3.91 ± 0.45a | 3.69 ± 0.25a | 0.0058 |
| Proline | 48.19 ± 3.42c | 73.69 ± 5.23a | 43.38 ± 3.81c | 54.68 ± 4.58b | 0.0012 |
| Acetic acid | 2.46 ± 0.16b | 2.61 ± 0.13ab | 2.1 ± 0.16c | 2.79 ± 0.15a | 0.0023 |
| Malic acid | 31.59 ± 5.28 | 38.25 ± 4.64 | 36.42 ± 2.47 | 34.29 ± 3.63 | 0.155 |
| Choline | 5.31 ± 0.21a | 4.28 ± 0.31b | 2.55 ± 0.24d | 3.86 ± 0.23c | 6.65E-04 |
| Phosphorylcholine | 1.07 ± 0.07b | 1.02 ± 0.16b | 2.07 ± 0.36a | 2.31 ± 0.13a | 0.0021 |

Note: Data are given as the mean ± std (standard deviation). Different letters within same row represent significant differences at the 0.05 level, and labels with the same letter are not significantly different at the level of 0.05, as determined by the S-N-K test.

**Electronic supplementary figure captions**

**ESI Figure. S1** ^1^H NMR (a) ,^13^C NMR (b) , ^1^H-^1^H COSY (c),^1^H-^13^C HSQC (d) and ^1^H-^13^C HMBC (e) NMR spectrum of isolated pure compounds. (A) vasicine;(B) vasicinone; (C) harmine;(D) harmaline.

**ESI Figure. S2** Aromatic region of 500MHz two-dimensional NMR spectra (TOCSY, HSQC) of *P Harmala* with peak assignments of alkaloids. (A) Aromatic region with expansion of δ 6.5-8.5 of ^1^H NMR spectra. a-8,6,7,5: H-8,6,7,5 of vasicinone, b-6,7,5,8: H-6,7,5,8 of vasicine, c-2: H-2 of harmine, d-1,2: H-1,2 of harmaline. (B) selected portion of TOCSY spectra with two spin system assignments. (C) selected portion of HSQC spectra showed the direct ^1^H/^13^C correlations, in the range of δ 6.5-8.5 of ^1^H and δ 90-140 of ^13^C.

**ESI Figure. S3** Chemical structural formula of alkaloids isolated from *P. harmala.*

**ESI Figure. S4** Validation result of PLS-DA for methanol extract samples by permutation. (A) May-August; (B) August-October; (C) October-December; (D) December-May.

A

a
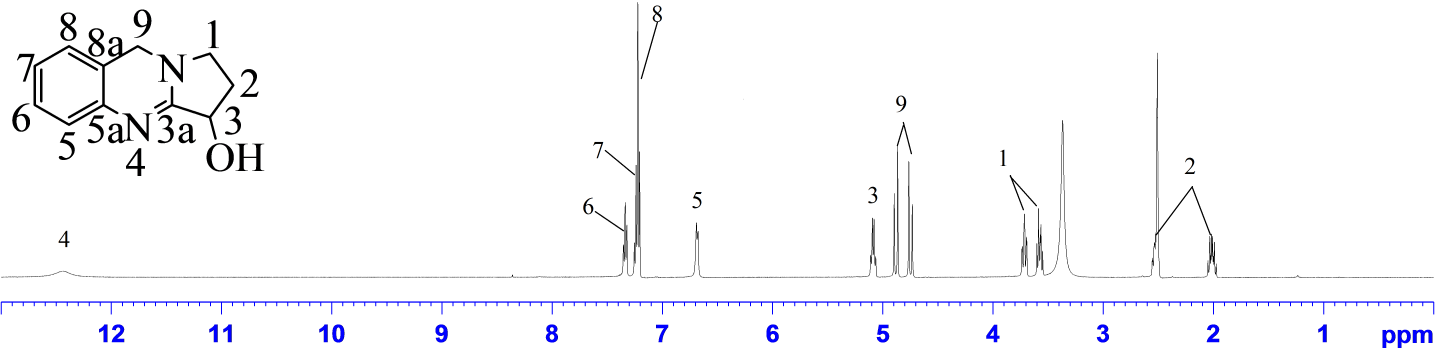


b

c

d

e

B

a


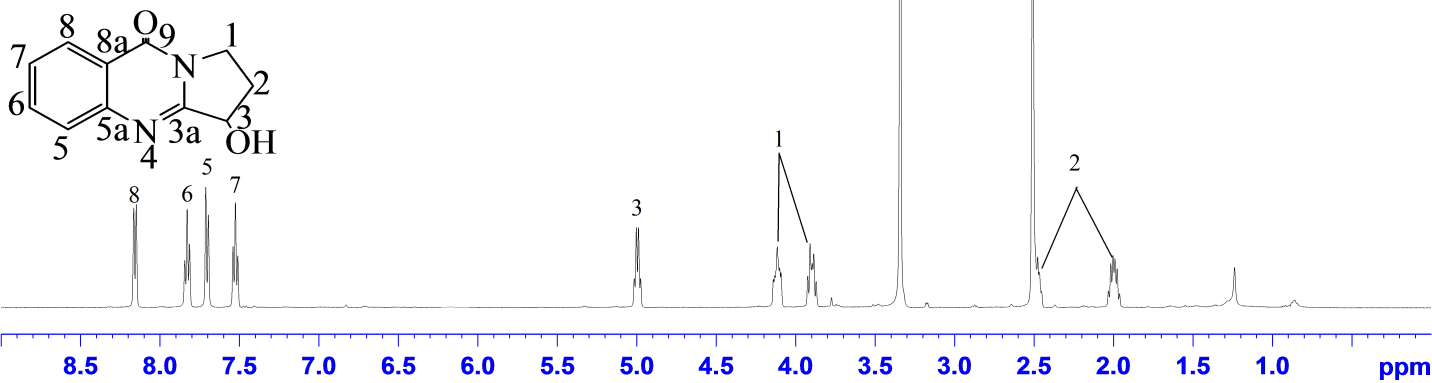


b

c

d

e

C

a


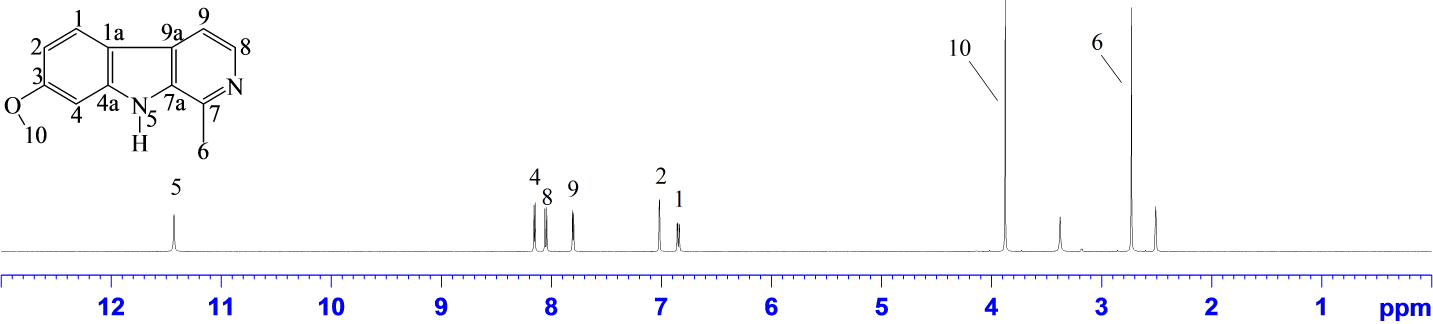


b

c

d

e

D

a


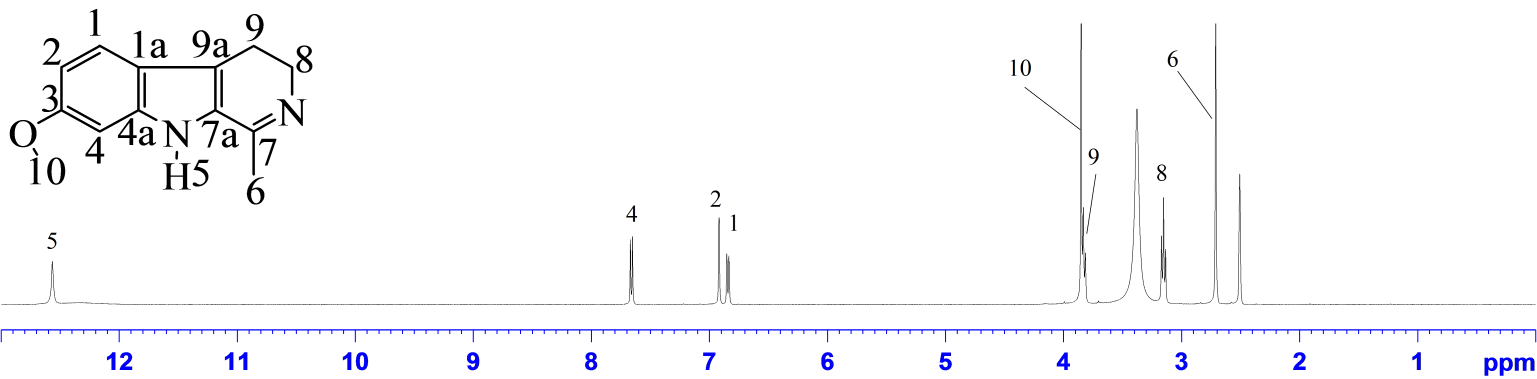


b

c

d

e

**ESI Figure. S1**


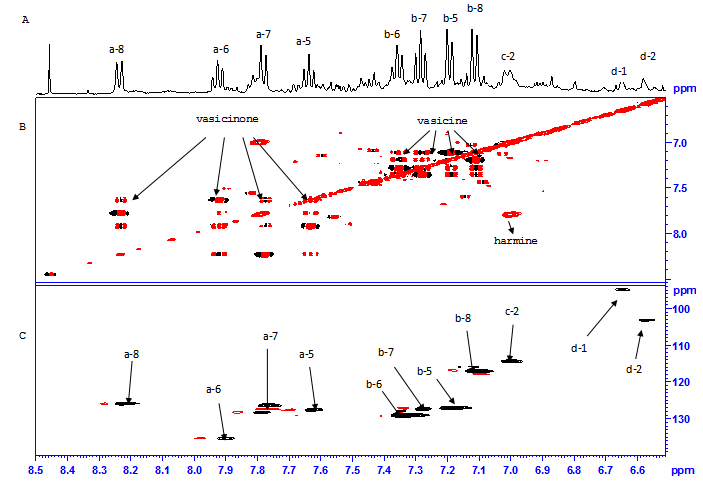


**ESI Figure. S2**


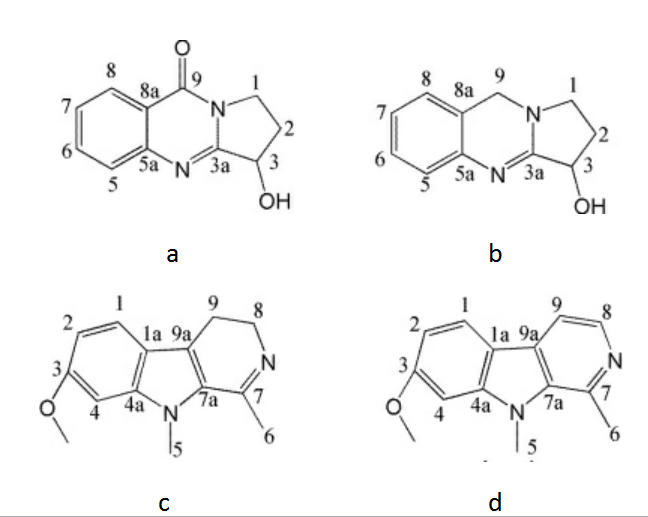


**ESI Figure. S3**

**ESI Figure. S4**
